# Metagenomic Analysis of Regularly Microwave-Treated and Untreated Domestic Kitchen Sponges

**DOI:** 10.3390/microorganisms8050736

**Published:** 2020-05-14

**Authors:** Susanne Jacksch, Jyothi Thota, Sudarshan Shetty, Hauke Smidt, Sylvia Schnell, Markus Egert

**Affiliations:** 1 Faculty of Medical and Life Sciences, Institute of Precision Medicine, Microbiology and Hygiene Group, Furtwangen University, 78054 Villingen-Schwenningen, Germany; Susanne.Jacksch@hs-furtwangen.de (S.J.); Jyothi.poda2@gmail.com (J.T.); 2 Laboratory of Microbiology, Wageningen University & Research, 6708 WE Wageningen, The Netherlands; sudarshan.shetty@wur.nl (S.S.); hauke.smidt@wur.nl (H.S.); 3 Institute of Applied Microbiology, Research Centre for BioSystems, Land Use, and Nutrition (IFZ), Justus-Liebig-University Giessen, 35392 Giessen, Germany; sylvia.schnell@umwelt.uni-giessen.de

**Keywords:** kitchen sponge, metagenomics, shotgun sequencing, microwave, kitchen hygiene

## Abstract

Kitchen sponges massively absorb and spread microorganisms, leading to contamination of kitchen appliances, surfaces, and food. Microwaving as an effective and widespread technique can rapidly reduce the microbial load of kitchen sponges. However, long-term effects of such treatments are largely unknown. Notably, it has been speculated that regularly applied domestic cleaning and disinfection may select for microbial communities with a higher pathogenic potential and/or malodorous properties. In this study, we distributed newly purchased polyurethane kitchen sponges to 20 participants, with the instruction to use them under normal household conditions for four weeks. Ten of the participants sanitized their sponges regularly by a standardized microwaving protocol, while the remaining ten sponges remained untreated. Metagenomic sequence data evaluation indicated that, in addition to bacteria, viruses, eukaryotes, and archaea were also part of the kitchen sponge microbiome. Comparisons of sanitized and untreated kitchen sponges indicated a trend towards a reduced structural microbial diversity while functional diversity increased. Microwave sanitization appeared to alter composition and metabolic properties of the microbial communities. Follow-up studies will have to show whether these changes are more positive or negative in terms of domestic hygiene, human health, and well-being.

## 1. Introduction

Within the domestic environment, kitchen utensils and surfaces are very frequently contaminated with microorganisms [1]. Used kitchen sponges contribute considerably to this microbial contamination [2]. The typical activity of wiping kitchen objects and surfaces following food preparation leads to the absorption of food residues and microorganisms [3]. Once contaminated, the constant nutrient-rich and humid environment offers ideal living conditions for microbial growth inside the sponge [4,5]. Several studies performed on kitchen sponges identified harmless environmental bacteria but also potential human pathogens as members of the microbial community [1,6,7,8,9,10]. Clearly, the use of a contaminated sponge might lead to the (re)contamination of kitchen appliances, surfaces and food, thereby increasing the risk of infections [8]. In addition, microbes might be spread to other areas of the household [11,12].

To control and reduce the microbial load in kitchen sponges, many different sanitization and cleaning procedures have been proposed [13,14], including microwaving or cleaning in a domestic dishwasher or washing machine [15,16,17,18]. Ikawa and colleagues [15] and Sharma and colleagues [17] evaluated the efficacy of different sponge cleaning methods by measuring the reduction of the microbial load in artificially contaminated samples. Both confirmed that microwave treatment is an effective and simple method to drastically reduce the bacterial load of kitchen sponges by five to seven log-scales [15,17].

Microwave radiation, as emitted by microwave ovens, can have different effects on microbial cells including thermal and nonthermal effects [19]. Thermal effects are caused by the absorption of microwave radiation, which causes the molecules inside the cell to vibrate and thus generate heat, which in turn causes denaturation of proteins and formation of aggregations in the cytoplasm [20,21]. On the other hand, nonthermal effects include changes in cell morphology and cell wall alterations or an enhanced protein or enzyme activity [8,21,22]. Still, the understanding of how microwaving affects microorganism is limited and a field of active research [20,23].

Many of the abovementioned studies examined the efficacy of sponge sanitization methods under controlled laboratory conditions. Interestingly, Ikawa and colleagues [15] showed that consumer-used sponges were much more difficult to disinfect, presumably due to the very high number of bacteria residing in used kitchen sponges. A recent study conducted by Cardinale and colleagues [6] reported local cell densities of up to 54 billion cells per cm^3^ as well as biofilm structures inside used kitchen sponges. Regularly sanitized kitchen sponges did not contain less bacteria than uncleaned ones, which is probably due to rapid recolonization of the sponge tissue by the (few) microorganisms surviving sanitization. The authors further suggested that regular cleaning might even select for higher proportions of potentially pathogenic and malodor-producing bacteria. Interestingly, there is a growing body of evidence that regular domestic cleaning and sanitization procedures might shape domestic microbial communities in a way that is non-beneficial for human health [24].

In the present study, we sought to address this hypothesis by analyzing kitchen sponges, which were either untreated or regularly sanitized by microwaving, through means of metagenomic shotgun sequencing. In addition to the structural characterization of the microbial community, functional capabilities were also examined for the influence of a regular microwave treatment. To the best of our knowledge, our study represents the first metagenomic study on used kitchen sponges, which probably represent the microbially most densely colonized inanimate objects in the domestic environment.

## 2. Materials and Methods

### 2.1. Study Design and Sample Collection

Twenty no-brand polyurethane sponges (~7 × 3 × 9 cm^3^) were bought in a store for household articles in Villingen-Schwenningen, Germany. The sponges were distributed to the 20 study participants, including students and academic staff of Furtwangen University as well as private household owners in the greater area of Freiburg (Germany) and Meiningen (Germany). The participants were instructed to use the kitchen sponges “as usual” under normal household conditions for a period of about four weeks. Ten, randomly chosen participants were advised to clean their kitchen sponges regularly two to three times a week by using a standardized microwaving protocol, which was based on previous studies [16,17,18]. Briefly, the kitchen sponge should first be soaked with tap water containing the participants’ own dishwashing detergent. Subsequently, the wet sponge should then be microwaved for 1 min at maximum wattage. After a short cooling phase, the kitchen sponge could be used again. After the study, the used (mostly wet) kitchen sponges were brought to the laboratory in a sterile bag and stored at -20 °C until further analysis.

A small survey was conducted voluntarily and anonymously to obtain information on the usage behavior by participants. Briefly, the kitchen sponges were used on average of 1 to 2 times per day for cleaning mainly dishes as well as kitchen surfaces. The household size ranged between 1 to 5 persons for the uncleaned sponge group and 1 to 7 for the cleaned ones. The maximum power of the microwaves used for sanitation was between 800 to 1200 watts.

### 2.2. DNA Purification

For DNA extraction, kitchen sponges were cut into halves and the corners and material from the middle part of each half were sampled with upper and lower parts (~2.5 cm^3^ in total) using sterile scissors. Cuts of each sponge half were pooled as one sample. Afterwards, DNA was extracted using the ZymoBIOMICS DNA Mini Kit (Zymo Research, Irvine, CA, USA) according to manufacturer´s specifications with some modifications. Briefly, after filtration, the Zymo-spin IV spin filter was repeatedly washed with 100 µL of DNAse free water for 3 or 4 times and the flow-through of the different washing steps was collected in separate tubes. To both the flow-through and the initial washing solution, binding buffer was added, and the solutions were successively applied onto a single Zymo-spin IIIC-Z Colum. Subsequently, the extracted DNA was eluted with 50 µL DNase/RNase free water and again purified using a Zymo-spin II column and 50 µL DNase/RNase-free water. After that, DNA concentration was determined with a Qubit 2.0 Fluorometer (Invitrogen, Carlsbad, CA, USA) using the Qubit dsDNA HS Assay Kit (Thermo Scientific, Waltham, MA, USA). To obtain a higher DNA concentration, all extracts stemming from the same sponge were combined to a single DNA extract of 50 µL by ethanol precipitation [25]. For library preparation, DNA concentration was measured again using the Qubit Fluorometer.

### 2.3. Library Preparation and Sequencing

The NEBNext Ultra II DNA Library Prep Kit (New England Biolabs, Ipswich, MA, USA) was used to create the library for shotgun sequencing, according to the manufacturer’s instructions. To get fragments with an estimated length of 150–300 bp, samples were incubated with the enzyme mix contained in the kit at 37 °C for 15–20 min depending on the input-DNA-concentration (if DNA concentration was <100 ng, incubation time was 15 min; if DNA concentration was >100 ng, incubation time was 20 min). The NEBNext Multiplex Oligos for Illumina (Index Primers Set 1 and 2) (New England Biolabs, Ipswich, MA, USA) were used to create the final DNA libraries. To add adapters to the fragmented DNA, the number of PCR cycles was selected depending on the amount of input DNA as specified in the manufacturer’s protocol. Fragment sizes and quality of the final DNA libraries were evaluated using an Agilent Bioanalyzer with the Agilent DNA 1000 Reagent kit (both Agilent, Santa Clara, CA, USA). Finally, all samples were sequenced on an Illumina MiSeq platform, using the Illumina MiSeq v2 Reagent Kit (Illumina, San Diego, CA, USA).

### 2.4. Bioinformatic Analyses

The unassembled reads were uploaded to the Metagenomic Rapid Annotation using Subsys-tems Technology (MG-RAST) pipeline v.4.0.3 for downstream analyses [26]. Taxonomic and functional profiles were generated using the MD5-based, nonredundant protein database (M5nr) [27] and REfSeq [28,29] for taxonomic classification and the SEED database [30] for functional profiling. To produce annotations, which were close matches to the reference database, the “representative hits classification” with an e-value cutoff of 5, a minimum identity cutoff of 80%, and a minimum alignment length cutoff of 50 bp was used.

Further processing of the data was done using R v.3.5.3 [31] and RStudio v.1.1.463 [32]. The main packages used in the analysis were vegan (v.2.5–6) [33] and phyloseq (v.1.26.1) [34]. The generated table of frequencies was normalized by rarefication to the smallest number of reads among the samples. For statistical analyses, Analysis of Variance (ANOVA) or Wilcoxon–Mann–Whitney-U tests for independent samples were applied. The resulting *p*-values were adjusted by Benjamin–Hochberg’s false discovery rate (FDR) [35]. Adjusted *p*-values < 0.05 were considered statistically significant. For alpha diversity analysis, the four most common diversity indices (Observed, Chao1, Shannon, and Simpson) were calculated and compared using ANOVA. For determination of beta diversity, taxonomic and functional profiles of samples were visualized by nonmetric multidimensional scaling (NMDS) using the Bray–Curtis distance measure. For further comparisons, Analysis of similarities (ANOSIM) and Permutational Multivariate Analysis of Variance Using Distance Matrices (ADONIS) were applied based on Bray–Curtis distances.

All sequence data were deposited at the MG-RAST server with the project ID mgp87011 (static link: https://www.mg-rast.org/linkin.cgi?project=mgp87011).

## 3. Results

### 3.1. DNA Extraction and Sequence Analysis

Regularly microwaved sponges yielded significantly lower, nevertheless still sufficient, amounts of genomic DNA for metagenomic analyses (Supplementary Table S1). After uploading the sequences to the MG-RAST server, the platform determined a total sequence quantity of 6,486,634 total sequences, with an average sequence length of about 150 bp. After quality control, performed by MG-RAST, 6,003,330 sequences remained (Supplementary Table S1). This corresponds to a loss of approximately 8% of the total sequences. It became apparent that the regularly sanitized kitchen sponges contained a lower quantity of sequences with a smaller average sequence length compared to the untreated kitchen sponges (Supplementary Table S1). Nevertheless, of the sequences remaining after quality control, about 2,248,216 reads could be annotated using the REfSeq database and, further, 625,777 reads could be annotated using the SEED subsystem classification. For further downstream analysis, a random subsampling was performed in both the taxonomic and functional analysis. This resulted in 38,471 sequences per sample for taxonomic analysis and 12,926 sequences per sample for functional profiles. Since unicellular organisms are of higher hygienic relevance, we focused our data analysis only on microorganisms.

### 3.2. Taxonomic Differences in Community Composition

Using the REfSeq database, the sequences were categorized from domain down to the genus level. After random subsampling, 97.0% of the sequences were assigned to the domain *Bacteria*, and 2.7% of all sequences were affiliated with viruses. Both, *Eukaryota* and *Archaea* showed lower relative abundances (<1%). A comparison between regularly sanitized und untreated kitchen sponges showed no significant differences at this level.

According to classification of MG-RAST, 42 phyla, 80 classes, 149 orders, 286 families, and 578 genera could be determined as members of the microbial community in used kitchen sponges. The most frequently occurring sequences belonged to the phyla *Proteobacteria* (86%), *Bacteroidetes* (7%), and *Actinobacteria* (4%) followed by unclassified viruses (3%). The most frequently identified genera were *Acinetobacter* (22%), *Enhydrobacter* (8%), *Agrobacterium* (6%), *Pseudomonas* (5%), and *Chrysobacterium* (2%) (Supplementary Figure S1). Interestingly, the bacterial community composition was significantly different between regularly microwaved and non-treated sponges (Table 1).

Microwaved sponges showed reduced relative abundances of *Bacteroidetes*, while in contrast, *Proteobacteria* were relatively increased. At the class level, *Gammaproteobacteria* increased in relative abundance due to microwaving, whereas relative abundances of *Betaproteobacteria*, *Flavobacteriia*, and *Sphingobacteriia* were decreased in treated sponges. At the order level, the relative abundances of *Pseudomonadales*, *Aeromonadales*, and *Enterobacteriales* were significantly higher in the microwaved sponges whereas the proportion of *Burkholderiales*, *Sphingobacteriales*, and *Flavobacteriales* was reduced. Similar changes were found at the family level. *Moraxellaceae*, *Pseudomonadaceae*, *Enterobacteriaceae* and *Aeromonadaceae* increased, while *Flavobacteriaceae*, *Brucellaceae* and *Alcaligenaceae* showed a reduction in their relative abundance in the regularly microwaved kitchen sponges. At the genus level, the relative abundances of genera such as *Acinetobacter*, *Citrobacter*, *Enterobacter*, *Escherichia*, and *Pseudomonas* were increased while *Bordetella*, *Chryseobacterium*, and *Ochrobactrum* were relatively less abundant in the treated sponges.

The most frequent type of viral DNA belonged to the genus *Microvirus*, which had a total mean relative abundance of 2.7%. However, only the order *Caudovirales* was significantly different between the untreated and sanitized sponges, with higher relative abundance in the latter. Only methanogens were found within the *Archaea* with *Methanococcoides* (total mean relative abundance 0.001%), *Methanoregula* (total mean relative abundance 0.01%), and *Methanosarcina* (total mean relative abundance 0.03%) being the most frequent genera. Notably, *Methanosarcina* showed a significantly higher relative abundance in regularly sanitized kitchen sponges when compared to the untreated sponges. Further significant differences in *Eukaryota*, *Archaea*, and viruses are presented in Table 2.

### 3.3. Differences in Community Structure

**Figure 1:**
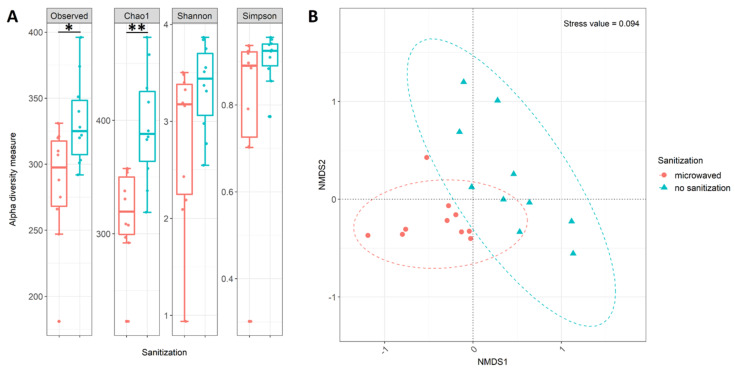
Taxonomic diversity analysis of used kitchen sponges calculated from the abundance table of all sequences at genus level: (**A**) Alpha diversity measures based on the four most common indices. Box plots show median as well as lower and upper quartiles. Each dot represents an individual sample. Whiskers represent minimum and maximum spread. Statistical analysis was done using ANOVA, and resulting FDR-corrected *p*-values are displayed as asterisks. The significant codes are *p* < 0.01 (**), *p* < 0.05 (*), and ( ) = not significant. (**B**) nonmetric multidimensional scaling (NMDS) plot using the Bray–Curtis distance measure of analyzed kitchen sponges. Color indicates sanitation treatment: regularly microwaved kitchen sponges (red) and untreated kitchen sponges (blue). Ellipses (dotted lines) represent the 95% confidence interval of each sanitization treatment.

To determine differences in diversity and community structure of the whole data set, the most common alpha diversity (Observed, Chao1, Shannon, and Simpson) indices were used (Figure 1A). All indices revealed differences between the microwaved and untreated used kitchen sponges regarding microbial diversity. More precisely, regularly microwaved sponges tended to have lower richness and diversity. Statistical analysis by ANOVA revealed that especially the indices Observed (ANOVA: *p* = 0.036) and Chao1 (ANOVA: *p* = 0.006) were significantly different, while the other alpha diversity indices (Shannon and Simpson) showed no significant influence of microwave treatment (ANOVA: *p*_Shannon_ = 0.105, *p*_Simpson_ = 0.119).

The beta diversity comparison suggested significant differences in microbial community composition between microwaved and untreated sponges. For this, NMDS ordination was used for graphical representation (Figure 1B). The NMDS plot revealed that the samples clustered according to their treatment, albeit with some overlay and considerable scattering within each cluster. However, a separation by treatment was confirmed through further statistical analysis using ANOSIM (ANOSIM: R = 0.471, *p* = 0.0001). Similar to ANOSIM, the ADONIS (*p* = 0.001, R^2^ = 0.278) analysis also indicated that the microbial composition of the two groups of sponges was statistically different.

### 3.4. Differences in Functional Annotation

**Figure 2:**
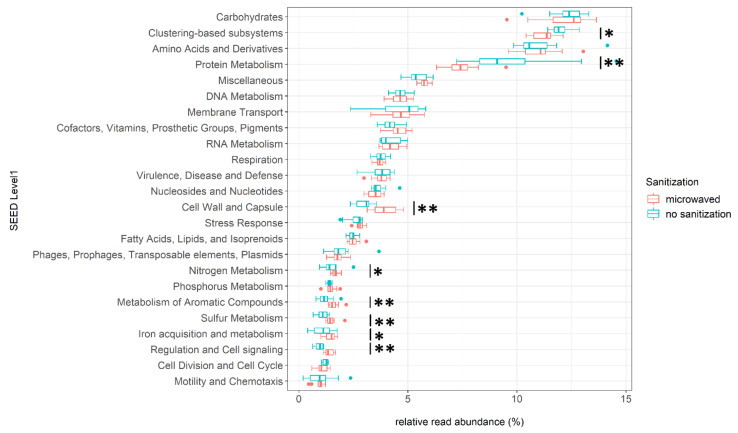
Functional profiles at SEED subsystem level 1 of sanitized and unsanitized used kitchen sponges: For better visualization, only the subsystems with a mean relative abundance greater than 1% are displayed. The boxes show median as well as lower and upper quartiles. Whiskers represent extremes outside upper and lower quartiles. The statistical comparison between microwaved and untreated kitchen sponges was done using Wilcoxon–Mann–Whitney-U tests for independent samples. Asterisks indicate subsystems that, in comparison, show a significant difference in relative abundance. The significant codes are *p* < 0.01 (**), *p* < 0.05 (*), and ( ) = not significant.

Next, for investigating the metabolic potential of the sponge communities, the uploaded sequences were compared to the hierarchical SEED database. Approximately 10% of the sequences could be attributed to a potential metabolic function. After random subsampling, the metagenomic reads could be categorized into three SEED categories. The determined 28 subsystems of level 1 could be broken down further into 187 SEED level 2 categories and 940 specific functions at SEED level 3. Based on the mean relative abundances across all samples, the most common metabolic categories were *carbohydrates* (12.2%), *clustering-based subsystems* (11.6%), *amino acids and derivatives* (11.0%), and *protein metabolism* (8.6%). Regularly microwaved and untreated sponges showed significant differences in potential metabolic functions (Figure 2).

For example, the relative abundances of genes for *regulation and cell signaling* (Wilcoxon: *p* = 0.001) increased from 0.9% in untreated kitchen sponges to 1.4% in microwaved sponges. The same applied to the subsystems for *cell wall and capsule* (Wilcoxon: *p* = 0.004) and *sulfur metabolism* (Wilcoxon: *p* = 0.006), where the relative abundances increased in treated kitchen sponges from 3% to 4% and from 1.1% to 1.5%, respectively.

Other SEED categories, such as *metabolism of aromatic compounds* (Wilcoxon: *p* = 0.006, untreated: 1.2%, microwaved: 1.6%), *nitrogen metabolism* (Wilcoxon: *p* = 0.020, untreated: 1.5%, microwaved: 1.7%), and *iron acquisition and metabolism* (Wilcoxon: *p* = 0.031, untreated: 1.1% microwaved: 1.5%) also showed significant differences.

The relative abundance of genes belonging to *protein metabolism* (Wilcoxon: *p* = 0.006) and *clustering-based subsystems* (Wilcoxon: *p* = 0.012) decreased from 9.8% to 7.5% and from 12.0% to 11.2%, respectively, in regularly microwaved kitchen sponges.

An overview of significantly differing SEED level 2 and 3 categories for all major SEED level 1 categories that showed significant differences between treated and untreated sponges (Figure 2) is provided in Supplementary Table S2.

**Figure 3:**
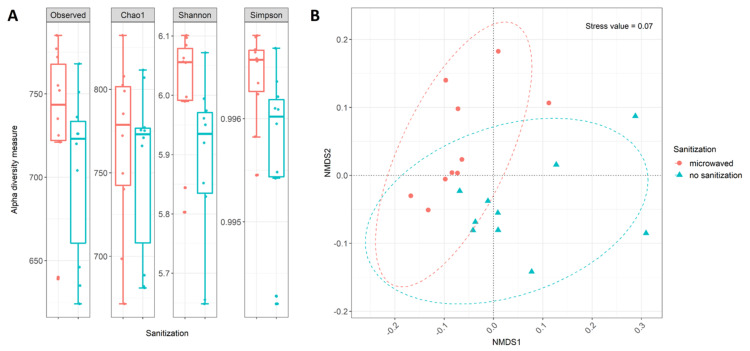
Diversity analysis of used kitchen sponges based on abundance table at SEED subsystem level 3: (**A**) Alpha diversity measures based one the four most common indices. Box plots show median as well as lower and upper quartiles. Each dot represents an individual sample. Whiskers represent minimum and maximum spread. (**B**) NMDS ordination for functional properties using the Bray–Curtis distance measures. Color indicates sanitation treatment: regularly microwaved sanitized kitchen sponges (red) and untreated kitchen sponges (blue). Dotted lines display ellipses, which represent the 95% confidence interval of each sanitization treatment.

Comparison of alpha and beta diversity based on annotated functions revealed differences between untreated and regularly sanitized kitchen sponges. Alpha diversity, for example, showed a trend towards an increased functional diversity in regularly microwaved sponges (Figure 3A). However, statistical analysis with ANOVA revealed that these differences were not significant. A comparison of the functional properties of SEED subsystem level 3 using NMDS displayed only minor differences between sanitized and untreated kitchen sponges with cluster overlaps and considerable scattering within each cluster (Figure 3B). A statistical analysis using ANOSIM (R = 0.21, *p* = 0.0028) confirmed the significance of the differences between the treatments. By using ADONIS (*p* = 0.006, R^2^ = 0.165), a significant influence of sanitization was also observed.

## 4. Discussion

Several previous studies addressed the microbial colonization of domestic kitchen sponges and showed that these widespread household items harbor a high bacterial load and a diverse bacterial population [1,6,7]. Some studies have demonstrated that different sanitization methods can significantly reduce this microbial load in the short term [16,17]. However, long-term effects of such sanitization methods on microbial community composition and particularly functionality are largely unknown. In this study, we analyzed ten regularly microwaved and ten untreated kitchen sponges by means of metagenomic shotgun sequencing to explore how regular disinfection affects microbial community composition and metabolic properties of the kitchen sponge community.

Our metagenomics analysis revealed that, in addition to prokaryotes (bacteria and archaea), viruses also represent a quantitatively important part of the kitchen sponge microbial community. The viruses found most frequently in this study were mainly bacteriophages, such as the genus *Microvirus* or the order *Caudovirales* [36]. Bacteriophages are one of the most common biological entities and are found wherever bacteria can grow [37]. Therefore, in line with the high bacterial abundance found in kitchen sponges, viral relative proportions were also expected to be abundant. More importantly, bacteriophages can have a considerable influence on the structure and function of microbial communities, such as species distribution [38].

In the present study, further members of the kitchen sponge community belonged to the domains of *Archaea* and *Eukaryota*. Regarding the domain *Eukaryota*, only microbial taxa were considered here, as sequences affiliated with multicellular organisms probably represent contaminating DNA from human, food, or other environmental sources, which are of minor hygienic relevance. Typical representatives of *Eukaryota* in the investigated kitchen sponges were different types of yeasts and molds. These have already been identified in other studies [5]. Within the archaeal domain, mainly methanogenic archaea were identified in kitchen sponges. Normally, this archaeal group needs an anoxic environment. However, it has been shown that especially the genus *Methanosarcina* contains species that can tolerate oxygen to a certain extent, which may be one reason why it represented the most abundant archaeal genus found in our study [39]. However, overall the relative abundance of *Archaea* and *Eukaryota* was low (<1%). Future studies with more detailed analyses of *Archaea* and *Eukaryota* in kitchen sponges will be crucial to identify whether these microbial groups merely represent minor important contaminations or if they are of hygienic relevance, too.

Microwave treatment of biological tissue causes thermal and nonthermal effects due to microwave radiation [23]. The data presented in this paper provides evidence that regularly microwaving also influences microbial community composition as well as functional profiles. Microbial diversity patterns of regularly microwaved and untreated sponges were clearly different. Microwaved kitchen sponges tended to have a lower alpha diversity of community composition than untreated sponges. Evaluating relative abundances at different taxonomic levels for sanitized and untreated kitchen sponges revealed that, in particular, *Gammaproteobacteria* benefited from a regular microwave treatment. Other classes, such as *Betaproteobacteria* or *Flavobacteriia*, decreased in their relative abundances. This trend implied a selection for certain bacterial populations by regular microwave sanitization, as was hypothesized by Cardinale et al. [6]. The genera that were relatively increased the most after microwave sanitization included *Acinetobacter*, *Klebsiella*, *Enterobacter*, and *Pseudomonas*. Further analyses are required to evaluate the pathogenic potential within these genera, since the use of the MG-RAST platform does not allow reliable taxa identification at species level.

Microwave radiation is also able to cause a variety of alterations within the metabolism of a cell and thereby might cause selective pressure on microorganisms [19]. The significant increase of genes affiliated with the subsystem *cell wall and capsule* observed in the regularly sanitized kitchen sponges might serve as an example. This subsystem includes genes for Gram-positive and Gram-negative cell wall components as well as capsular and extracellular polysaccharides and may be a hint for adaptive alterations in biofilm formation. It was shown that thermal stress can weaken the integrity of a microbial biofilm, which is therefore more easily sheared off from a contaminated device [40]. It should also be noted that biofilm formation is a vital characteristic for microbial survival under extreme conditions, such as heat stress [41]. The ability to form biofilms might be one cause for the positive selection of *Gammaproteobacteria*, since the genera detected at increased relative abundance in microwaved sponges here, such as *Acinetobacter* and *Pseudomonas*, are very well-known biofilm-formers [42,43].

In addition, increased relative abundances of genes belonging to the metabolism of sulfur, iron, or aromatic compounds might also be due to the higher shares of *Gammaproteobacteria*, as they possess a very versatile metabolism with respect to these substrates [44].

Since sulfur is an important component of many malodorous substances, such as H_2_S, the observed relative increase in genes affiliated with sulfur metabolism might be carefully interpreted as a higher potential towards malodor formation. However, this hypothesis clearly needs to be corroborated by physiological data, including direct measurement of malodorous substances as well as environmental parameters, such as oxygen or pH, which influence microbial activities in this respect [45,46,47].

Despite the noticeable variability within each sanitization group, likely caused by different environmental conditions in the participants’ households, there was a significant difference between them. Follow-up studies should therefore include more samples and should be more standardized. With our experimental design, it cannot be ruled out that the observed effects not only were caused by the microwave treatment but also might result from other factors differing between the two groups of participants. It is therefore important, for further studies, to collect more user and usage metadata and to define the experimental conditions (microwaving parameters, dishwashing detergent used for soaking, etc.) as precisely as possible in order to ensure a better comparability and to verify the hypotheses put forward here. Follow-up studies should particularly address the relevance of microwave-induced changes in microbial community structure and function for domestic hygiene, human health, and well-being. In our view, pathogenic potential, biofilm formation capacity, and sulfur metabolism/malodor production appear of particular interest in this context.

## Figures and Tables

**Table 1:** Significant differences in bacterial community composition between regularly microwaved and untreated kitchen sponges: Relative abundances of taxa with a mean relative abundance of more than one percent per treatment are shown. Wilcoxon–Mann–Whitney-U tests for independent samples were performed to identify statistically significant differences. False discovery rate (FDR)-corrected *p*-values are displayed as well. Color visualizes higher or lower relative abundances and goes from green (0%) over yellow to red (100%).

Taxonomy Level	Organism	Microwaved (%)	No Treatment (%)	*p*-Values
**Phylum**	*Bacteroidetes*	2.0	12.9	0.009
	*Proteobacteria*	92.0	79.2	0.013
**Class**	*Sphingobacteriia*	0.2	1.2	0.008
	*Flavobacteriia*	1.6	11.2	0.009
	*Gammaproteobacteria*	71.3	33.6	0.009
	*Betaproteobacteria*	2.7	7.5	0.031
**Order**	*Sphingobacteriales*	0.2	1.2	0.006
	*Flavobacteriales*	1.5	11.2	0.011
	*Aeromonadales*	2.0	0.2	0.006
	*Enterobacteriales*	19.2	3.7	0.006
	*Pseudomonadales*	48.1	24.1	0.035
	*Burkholderiales*	2.4	7.2	0.035
**Family**	*Sphingobacteriaceae*	0.2	1.1	0.011
	*Flavobacteriaceae*	1.5	11.0	0.018
	*Aeromonadaceae*	2.0	0.2	0.011
	*Enterobacteriaceae*	19.2	3.7	0.011
	*Moraxellaceae*	40.6	21.7	0.046
	*Pseudomonadaceae*	7.6	2.4	0.023
	*Alcaligenaceae*	0.3	3.6	0.023
	*Brucellaceae*	0.6	4.9	0.031
**Genus**	*Chryseobacterium*	0.5	3.6	0.011
	*Riemerella*	0.2	1.1	0.015
	unclassified *(Flavobacteriaceae)*	0.3	4.8	0.011
	*Aeromonas*	1.9	0.2	0.011
	*Citrobacter*	2.7	0.3	0.011
	*Salmonella*	1.4	0.3	0.011
	*Escherichia*	2.0	0.4	0.011
	*Enterobacter*	5.8	0.9	0.011
	*Klebsiella*	5.7	1.4	0.018
	*Acinetobacter*	34.6	8.4	0.011
	*Pseudomonas*	7.4	2.4	0.020
	*Bordetella*	0.1	1.0	0.011
	*Achromobacter*	0.2	2.6	0.024
	*Brucella*	0.2	2.0	0.024
	*Ochrobactrum*	0.5	2.9	0.033
	*Caulobacter*	0.4	4.4	0.043

**Table 2:** Significant differences in eukaryotic, archaeal and viral community composition (according to classification of Metagenomic Rapid Annotation using Subsystems Technology (MG-RAST)) between regularly microwaved and untreated kitchen sponges: Mean relative abundances of taxa per treatment are shown. Wilcoxon–Mann–Whitney-U tests for independent samples were performed to identify statistically significant differences. FDR-corrected *p*-values are displayed as asterisks (*p* < 0.01 (**) and *p* < 0.05 (*)).

Kingdom	*Eukaryota*				*Archaea*				Viruses			
Taxonomy Level	Organism	Microwaved (%)	No Treatment (%)	Sig.	Organism	Microwaved (%)	No Treatment (%)	Sig.	Organism	Microwaved (%)	NoTreatment (%)	Sig.
Class	unclassified (*Eukaryota*)	0.000	0.004	*	none	*-*	-	-	none	*-*	-	-
Order	unclassified (*Eukaryota*)	0.000	0.004	*	*Methanosarcinales*	0.008	0.000	*	*Caudovirales*	0.064	0.025	*
Family	unclassified (*Eukaryota*)	0.000	0.004	*	*Methanosarcinaceae*	0.008	0.000	*	*Myoviridae*	0.028	0.005	*
	*Metschnikowiaceae*	0.003	0.000	*					*Podoviridae*	0.010	0.001	*
									*Siphoviridae*	0.027	0.018	*
Genus	*Acanthamoeba*	0.000	0.004	*	*Methanosarcina*	0.005	0.000	*	Lambda-like viruses	0.008	0.003	*
	*Clavispora*	0.003	0.000	*					P2-like viruses	0.027	0.002	*
	*Aspergillus*	0.000	0.004	*					T7-like viruses	0.006	0.001	*
									unclassified (*Podoviridae*)	0.003	0.000	*

## Supplementary Materials

The following are available online at https://www.mdpi.com/2076-2607/8/5/736/s1. Figure S1: Relative counts of regularly microwaved and un-treated used kitchen sponges summarized at phylum and genus level, Table S1: Summary statistics of the sequence data of unsanitized and regularly sanitized used kitchen sponges uploaded to MG-RAST, Table S2: Overview of the significantly differing level 3 functions from the level1 SEED subsystems that were found to be significantly different between treated and untreated sponges.
