# Supplementary material for: Metagenomic Analysis of Regularly Microwave-Treated and Untreated Domestic Kitchen Sponges
Source: Microorganisms. 2020 May 14;8(5):736. doi: 10.3390/microorganisms8050736 (PMC7284620; doi:10.3390/microorganisms8050736)
Supplement: Supplementary file 1 [file microorganisms-08-00736-s001.zip › Supp/Table_S1.docx]

**Table S1.** Summary statistics of the sequence data of unsanitized and regularly sanitized used kitchen sponges uploaded to MG-RAST. Values correspond to the uploaded sequence counts before and after the quality control performed by the MG-RAST platform. The individual sample names, the names which were automatically assigned by the platform, and DNA concentrations after extraction are shown. NCS = Nonclean (untreated) sponges, CS = Clean (microwaved) sponges. *(Continuation of table S1 on the next page).*

| Sample names | MG-RAST ID | DNA concentration (ng/µl) | Upload: bp Count (bp) | Upload: Sequences Count | Upload: Mean Sequence Length (bp) | Upload: Mean GC percent (%) | Artificial Duplicate Reads: Sequence Count | Post QC: bp Count (bp) | Post QC: Sequences Count | Post QC: Mean Sequence Length (bp) | Post QC: Mean GC percent (%) |
| --- | --- | --- | --- | --- | --- | --- | --- | --- | --- | --- | --- |
| NCS1 | [mgm4814162.3](https://www.mg-rast.org/mgmain.html?mgpage=overview&metagenome=mgm4814162.3) | 78.8 | 60693454 | 322433 | 188 ± 68 | 59 ± 12 | 2759 | 58128794 | 308121 | 189 ± 64 | 59 ± 11 |
| NCS2 | [mgm4814166.3](https://www.mg-rast.org/mgmain.html?mgpage=overview&metagenome=mgm4814166.3) | 172.0 | 77009275 | 402149 | 191 ± 73 | 51 ± 15 | 4498 | 73652597 | 383180 | 192 ± 69 | 51 ± 14 |
| NCS3 | [mgm4814165.3](https://www.mg-rast.org/mgmain.html?mgpage=overview&metagenome=mgm4814165.3) | 135.0 | 59368002 | 334335 | 178 ± 67 | 58 ± 14 | 3107 | 56848635 | 318504 | 178 ± 62 | 58 ± 12 |
| NCS4 | [mgm4814169.3](https://www.mg-rast.org/mgmain.html?mgpage=overview&metagenome=mgm4814169.3) | 137.0 | 79182405 | 499344 | 159 ± 69 | 58 ± 14 | 4769 | 74026966 | 462660 | 160 ± 61 | 59 ± 12 |
| NCS5 | [mgm4814155.3](https://www.mg-rast.org/mgmain.html?mgpage=overview&metagenome=mgm4814155.3) | 20.0 | 63894745 | 417639 | 153 ± 73 | 63 ± 14 | 3778 | 58873704 | 374611 | 157 ± 64 | 65 ± 9 |
| NCS6 | [mgm4814167.3](https://www.mg-rast.org/mgmain.html?mgpage=overview&metagenome=mgm4814167.3) | 76.6 | 57051535 | 383460 | 149 ± 73 | 55 ± 16 | 4047 | 53042435 | 346158 | 153 ± 65 | 56 ± 14 |
| NCS7 | [mgm4814156.3](https://www.mg-rast.org/mgmain.html?mgpage=overview&metagenome=mgm4814156.3) | 30.5 | 57751644 | 374210 | 154 ± 70 | 46 ± 14 | 6417 | 54339346 | 346000 | 157 ± 65 | 46 ± 13 |
| NCS8 | [mgm4814168.3](https://www.mg-rast.org/mgmain.html?mgpage=overview&metagenome=mgm4814168.3) | 41.3 | 59015511 | 380357 | 155 ± 80 | 60 ± 15 | 5595 | 55039657 | 345349 | 159 ± 75 | 60 ± 14 |
| NCS9 | [mgm4814164.3](https://www.mg-rast.org/mgmain.html?mgpage=overview&metagenome=mgm4814164.3) | 10.4 | 53311028 | 343166 | 155 ± 74 | 47 ± 13 | 4542 | 49905315 | 317664 | 157 ± 69 | 47 ± 12 |
| NCS10 | [mgm4814159.3](https://www.mg-rast.org/mgmain.html?mgpage=overview&metagenome=mgm4814159.3) | 17.8 | 62916282 | 376845 | 167 ± 70 | 58 ± 14 | 3848 | 59294637 | 353553 | 168 ± 63 | 59 ± 12 |
| **Total sum of sequences** |  |  |  | **3833938** |  |  |  |  | **3555800** |  |  |

| Sample names | MG-RAST ID | DNA concentration (ng/µl) | Upload: bp Count (bp) | Upload: Sequences Count | Upload: Mean Sequence Length (bp) | Upload: Mean GC percent (%) | Artificial Duplicate Reads: Sequence Count | Post QC: bp Count (bp) | Post QC: Sequences Count | Post QC: Mean Sequence Length (bp) | Post QC: Mean GC percent (%) |
| --- | --- | --- | --- | --- | --- | --- | --- | --- | --- | --- | --- |
| CS1 | [mgm4814170.3](https://www.mg-rast.org/mgmain.html?mgpage=overview&metagenome=mgm4814170.3) | 0.3 | 36049299 | 264149 | 136 ± 76 | 57 ± 12 | 2008 | 33111553 | 241972 | 137 ± 70 | 58 ± 11 |
| CS2 | [mgm4814153.3](https://www.mg-rast.org/mgmain.html?mgpage=overview&metagenome=mgm4814153.3) | 1.1 | 25463066 | 170649 | 149 ± 78 | 47 ± 11 | 2282 | 23672227 | 159159 | 149 ± 74 | 47 ± 11 |
| CS3 | [mgm4814160.3](https://www.mg-rast.org/mgmain.html?mgpage=overview&metagenome=mgm4814160.3) | 6.2 | 50532205 | 348155 | 145 ± 64 | 50 ± 13 | 4202 | 47185773 | 328629 | 144 ± 57 | 50 ± 12 |
| CS4 | [mgm4814154.3](https://www.mg-rast.org/mgmain.html?mgpage=overview&metagenome=mgm4814154.3) | 11.2 | 36585136 | 280361 | 130 ± 58 | 50 ± 13 | 2660 | 34588151 | 264008 | 131 ± 53 | 50 ± 12 |
| CS5 | [mgm4814158.3](https://www.mg-rast.org/mgmain.html?mgpage=overview&metagenome=mgm4814158.3) | 16.7 | 49048217 | 333052 | 147 ± 67 | 57 ± 12 | 5138 | 46355168 | 313885 | 148 ± 63 | 57 ± 11 |
| CS6 | [mgm4814172.3](https://www.mg-rast.org/mgmain.html?mgpage=overview&metagenome=mgm4814172.3) | 3.4 | 31140581 | 231963 | 134 ± 81 | 59 ± 13 | 2364 | 28582320 | 203917 | 140 ± 76 | 59 ± 11 |
| CS7 | [mgm4814171.3](https://www.mg-rast.org/mgmain.html?mgpage=overview&metagenome=mgm4814171.3) | 1.1 | 30102361 | 241427 | 125 ± 77 | 62 ± 10 | 1852 | 26003971 | 216773 | 120 ± 67 | 62 ± 9 |
| CS8 | [mgm4814163.3](https://www.mg-rast.org/mgmain.html?mgpage=overview&metagenome=mgm4814163.3) | 4.5 | 52595670 | 367736 | 143 ± 63 | 54 ± 14 | 3624 | 49331207 | 340901 | 145 ± 57 | 54 ± 13 |
| CS9 | [mgm4814161.3](https://www.mg-rast.org/mgmain.html?mgpage=overview&metagenome=mgm4814161.3) | 2.1 | 25460363 | 187646 | 136 ± 77 | 54 ± 12 | 2030 | 23076072 | 172703 | 134 ± 70 | 54 ± 12 |
| CS10 | [mgm4814157.3](https://www.mg-rast.org/mgmain.html?mgpage=overview&metagenome=mgm4814157.3) | 0.9 | 32681894 | 227558 | 144 ± 82 | 55 ± 13 | 2009 | 29195910 | 205583 | 142 ± 76 | 55 ± 12 |
| **Total sum of sequences** |  |  |  | **2652696** |  |  |  |  | **2447530** |  |  |
