# Supplementary material for: Metagenomic Analysis of Regularly Microwave-Treated and Untreated Domestic Kitchen Sponges
Source: Microorganisms. 2020 May 14;8(5):736. doi: 10.3390/microorganisms8050736 (PMC7284620; doi:10.3390/microorganisms8050736)
Supplement: Supplementary file 1 [file microorganisms-08-00736-s001.zip › Supp/Table_S2.docx]

**Table S2**. Overview of the significantly differing level 3 functions from the level1 SEED subsystems that were found to be significantly different between treated and untreated sponges. Mean relative abundances of functions per treatment are shown, as well as FDR corrected p-values. Wilcoxon-Mann-Whitney-U tests for independent samples were performed to identify significant differences. Color visualizes higher or lower relative abundances and goes from green (= 0%) over yellow to red (= 1,1%). *(Continuation of the table on the following pages)*

| Level1 | Level2 | Level3 | Microwaved (%) | No treatment (%) | P-values |
| --- | --- | --- | --- | --- | --- |
| Protein Metabolism | Protein biosynthesis | Ribosome LSU bacterial | 0.582 | 1.065 | 0.006 |
|  |  | Ribosome SSU bacterial | 0.436 | 0.811 | 0.006 |
|  |  | Translation elongation factors bacterial | 0.294 | 0.416 | 0.010 |
|  |  | Translation elongation factors eukaryotic and archaeal | 0.015 | 0.022 | 0.029 |
|  |  | tRNA aminoacylation, Ser | 0.008 | 0.015 | 0.011 |
|  |  | tRNA aminoacylation, Lys | 0.101 | 0.192 | 0.011 |
|  |  | tRNA aminoacylation, Asp and Asn | 0.114 | 0.193 | 0.021 |
|  |  | tRNA aminoacylation, Arg | 0.116 | 0.094 | 0.037 |
|  |  | Universal GTPases | 0.397 | 0.816 | 0.010 |
|  | Protein processing and modification | Ribosomal protein S12p Asp methylthiotransferase | 0.016 | 0.036 | 0.012 |
|  |  | Ribosomal protein S5p acylation | 0.046 | 0.071 | 0.041 |
|  |  | Signal peptidase | 0.011 | 0.005 | 0.046 |
|  | Protein folding | Periplasmic disulfide interchange | 0.036 | 0.017 | 0.029 |
|  | Protein degradation | Proteolysis in bacteria, ATP-dependent | 0.439 | 0.569 | 0.010 |
|  | Selenoproteins | Selenocysteine metabolism | 0.078 | 0.039 | 0.021 |
| Cell Wall and Capsule | Gram-Negative cell wall components | Inner membrane protein YhjD and conserved cluster involved in LPS biosynthesis | 0.054 | 0.008 | 0.007 |
|  |  | Lipid A modifications | 0.092 | 0.026 | 0.008 |
|  |  | Outer membrane | 0.122 | 0.056 | 0.012 |
|  |  | Lipopolysaccharide assembly | 0.332 | 0.200 | 0.017 |
|  |  | Major Outer Membrane Proteins | 0.159 | 0.091 | 0.033 |
|  |  | KDO2-Lipid A biosynthesis | 0.354 | 0.313 | 0.037 |
|  | Capsular and extracellular polysacchrides | Colanic acid biosynthesis | 0.084 | 0.021 | 0.010 |
|  |  | Alginate metabolism | 0.142 | 0.091 | 0.017 |
|  |  | Capsular Polysaccharides Biosynthesis and Assembly | 0.032 | 0.008 | 0.019 |
|  |  | YjbEFGH Locus Involved in Exopolysaccharide Production | 0.021 | 0.001 | 0.028 |
|  |  | Capsular Polysaccharide (CPS) of Campylobacter | 0.000 | 0.004 | 0.040 |
|  | NULL | Murein Hydrolases | 0.311 | 0.194 | 0.024 |
|  |  | Peptidoglycan Crosslinking of Peptide Stems | 0.029 | 0.006 | 0.035 |
|  |  | Peptidoglycan biosynthesis--gjo | 0.169 | 0.122 | 0.037 |
| Nitrogen Metabolism | NULL | Nitrosative stress | 0.083 | 0.026 | 0.013 |
|  |  | Nitrate and nitrite ammonification | 0.733 | 0.600 | 0.017 |
|  |  | Nitric oxide synthase | 0.003 | 0.026 | 0.028 |
|  |  | Dissimilatory nitrite reductase | 0.000 | 0.002 | 0.040 |
| Metabolism of Aromatic Compounds | Peripheral pathways for catabolism of aromatic compounds | Benzoate degradation | 0.194 | 0.053 | 0.006 |
|  | Metabolism of central aromatic intermediates | Protocatechuate branch of beta-ketoadipate pathway | 0.189 | 0.091 | 0.033 |
|  |  | N-heterocyclic aromatic compound degradation | 0.006 | 0.031 | 0.039 |
|  | Anaerobic degradation of aromatic compounds | Hydroxyaromatic decarboxylase family | 0.014 | 0.001 | 0.025 |
|  |  | Anaerobic benzoate metabolism | 0.091 | 0.137 | 0.046 |
|  | Metabolism of central aromatic intermediates | 4-Hydroxyphenylacetic acid catabolic pathway | 0.057 | 0.026 | 0.049 |
| Sulfur Metabolism | Organic sulfur assimilation | Alkanesulfonate assimilation | 0.303 | 0.200 | 0.011 |
|  | NULL | Galactosylceramide and Sulfatide metabolism | 0.060 | 0.026 | 0.019 |
|  |  | Thioredoxin-disulfide reductase | 0.255 | 0.138 | 0.033 |
|  |  | Sulfur oxidation | 0.010 | 0.016 | 0.041 |
| Iron acquisition and metabolism | Siderophores | Siderophore Enterobactin | 0.129 | 0.029 | 0.008 |
|  |  | Siderophore assembly kit | 0.093 | 0.044 | 0.026 |
|  | NULL | Heme, hemin uptake and utilization systems in GramNegatives | 0.133 | 0.094 | 0.012 |
|  |  | Heme, hemin uptake and utilization systems in GramPositives | 0.018 | 0.008 | 0.013 |
|  |  | Ferrous iron transporter EfeUOB, low-pH-induced | 0.046 | 0.011 | 0.037 |
| Regulation and Cell signaling  Regulation and Cell signaling | Programmed Cell Death and Toxin-antitoxin Systems | Murein hydrolase regulation and cell death | 0.096 | 0.034 | 0.006 |
|  | Regulation of virulence | A conserved operon linked to TyrR and possibly involved in virulence | 0.015 | 0.002 | 0.017 |
|  | Quorum sensing and biofilm formation | Autoinducer 2 (AI-2) transport and processing (lsrACDBFGE operon) | 0.061 | 0.012 | 0.033 |
|  | NULL | Orphan regulatory proteins | 0.224 | 0.123 | 0.006 |
|  |  | DNA-binding regulatory proteins, strays | 0.194 | 0.097 | 0.010 |
|  |  | Rcs phosphorelay signal transduction pathway | 0.034 | 0.010 | 0.013 |
|  |  | The Chv regulatory system of Alphaproteobacteria | 0.025 | 0.047 | 0.021 |
|  |  | Trans-envelope signaling system VreARI in Pseudomonas | 0.000 | 0.009 | 0.025 |
|  |  | cAMP signaling in bacteria | 0.105 | 0.073 | 0.033 |
|  |  | Sex pheromones in Enterococcus faecalis and other Firmicutes | 0.019 | 0.005 | 0.049 |
| Clustering-based subsystems | Probably organic hydroperoxide resistance related hypothetical protein | CBSS-269482.1.peg.1294 | 0.036 | 0.013 | 0.011 |
|  | Molybdopterin oxidoreductase | CBSS-269799.3.peg.2220 | 0.006 | 0.019 | 0.024 |
|  | Hypothetical Related to Dihydroorate Dehydrogenase | Hypothetical Related to Dihydroorotate dehydrogenase | 0.012 | 0.050 | 0.013 |
|  | Lysine, threonine, methionine, and cysteine | YeiH | 0.137 | 0.063 | 0.017 |
|  | Probably Ybbk-related hypothetical membrane proteins | CBSS-316057.3.peg.659 | 0.060 | 0.029 | 0.021 |
|  | Tricarboxylate transporter | CBSS-49338.1.peg.459 | 0,099 | 0,213 | 0,029 |
|  | Putative asociate of RNA polymerase sigma-54 factor rpoN | CBSS-316057.3.peg.1308 | 0,053 | 0,159 | 0,033 |
|  | TldD cluster | CBSS-354.1.peg.2917 | 0,111 | 0,060 | 0,041 |
|  | Cell Division | CBSS-393130.3.peg.794 | 0,033 | 0,067 | 0,041 |
|  | CRISPRs and associated hypotheticals | CBSS-216592.1.peg.3534 | 0,023 | 0,013 | 0,046 |
|  | Protein export? | CBSS-393121.3.peg.2760 | 0,162 | 0,261 | 0,046 |
|  | Fatty acid metabolic cluster | COG1399 | 0,255 | 0,418 | 0,046 |
|  | NULL | CBSS-138119.3.peg.2719 | 0,032 | 0,128 | 0,006 |
|  |  | CBSS-350688.3.peg.1509 | 0,498 | 0,801 | 0,006 |
|  |  | CBSS-83332.1.peg.3803 | 0,002 | 0,026 | 0,006 |
|  |  | CBSS-83333.1.peg.946 | 0,122 | 0,039 | 0,006 |
|  |  | Cluster with phosphopentomutase paralog | 0,018 | 0,001 | 0,008 |
|  |  | EC699-706 | 0,067 | 0,022 | 0,010 |
|  |  | RNA modification and chromosome partitioning cluster | 0,069 | 0,125 | 0,010 |
|  |  | DNA gyrase subunits | 0,101 | 0,234 | 0,012 |
|  |  | Ribonucleotide reductase cluster | 0,067 | 0,145 | 0,013 |
|  |  | CBSS-214092.1.peg.3450 | 0,016 | 0,002 | 0,017 |
|  |  | tRNA-methylthiotransferase containing cluster | 0,090 | 0,050 | 0,017 |
|  |  | Cluster co-expressed with butyrate metabolism cluster | 0,060 | 0,029 | 0,019 |
|  |  | Conserved cluster in Enterobacteriaceae downstream from YqjA, a DedA family protein | 0,016 | 0,008 | 0,021 |
|  |  | Conserved cluster around acetyltransferase YpeA in Enterobacteria | 0,018 | 0,001 | 0,023 |
|  |  | CBSS-235.1.peg.567 | 0,283 | 0,325 | 0,024 |
|  |  | EC49-61 | 0,203 | 0,132 | 0,024 |
|  |  | PFGI-1-like cluster 2 | 0,015 | 0,003 | 0,028 |
|  |  | Glutaredoxin 3 containing cluster 2 | 0,003 | 0,010 | 0,028 |
|  |  | CBSS-176299.4.peg.1996B | 0,117 | 0,197 | 0,029 |
|  |  | CBSS-160492.1.peg.550 | 0,059 | 0,023 | 0,037 |
|  |  | CBSS-316407.3.peg.2816 | 0,025 | 0,014 | 0,041 |
|  |  | CBSS-393121.3.peg.1913 | 0,063 | 0,121 | 0,041 |
|  |  | USS-DB-7 | 0,070 | 0,029 | 0,041 |
|  |  | CBSS-211586.1.peg.3133 | 0,063 | 0,038 | 0,046 |
