# Supplementary figures and images for: Metagenomic Analysis of Regularly Microwave-Treated and Untreated Domestic Kitchen Sponges
Source: Microorganisms. 2020 May 14;8(5):736. doi: 10.3390/microorganisms8050736 (PMC7284620; doi:10.3390/microorganisms8050736)

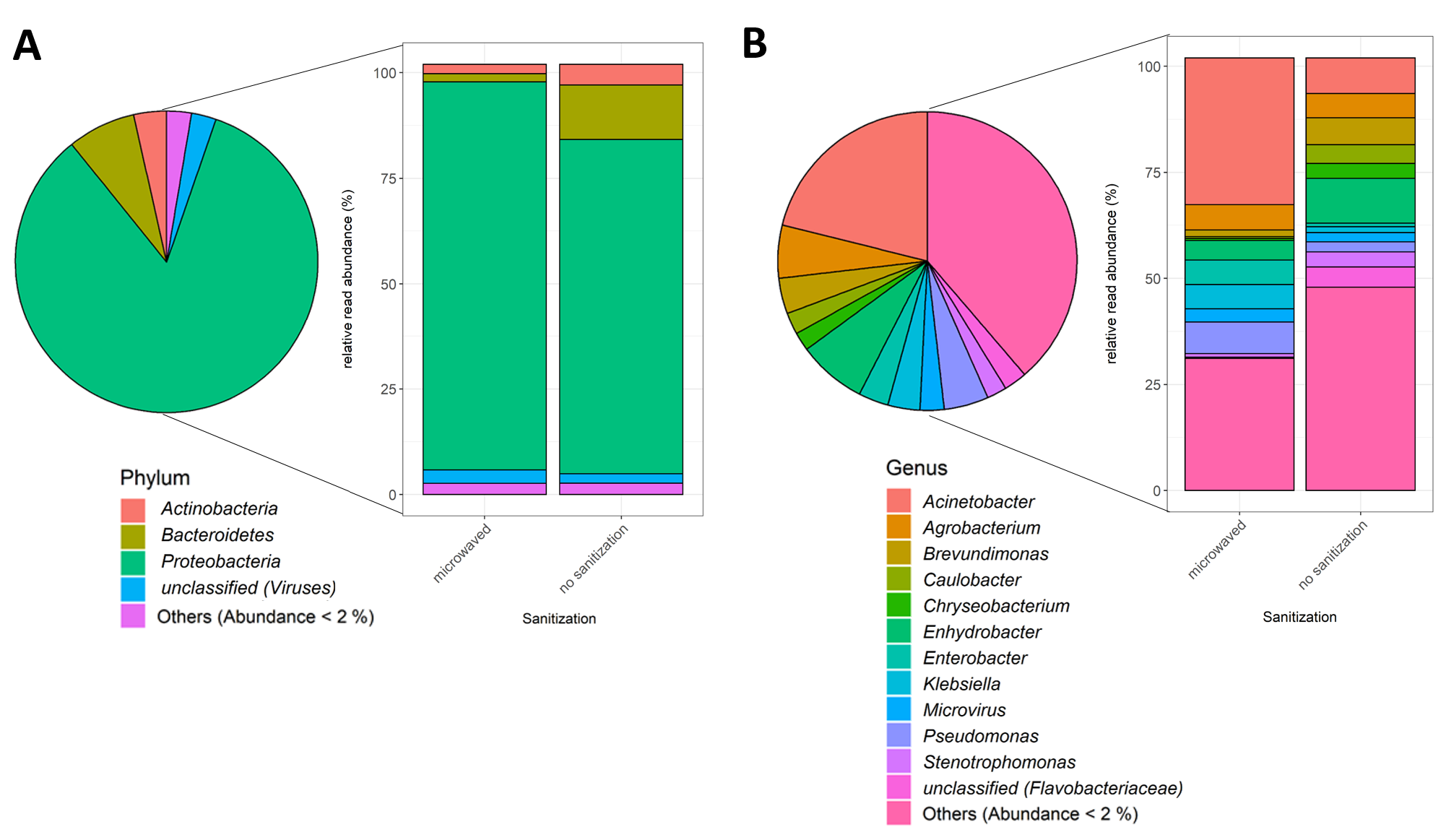

Supplement: Supplementary file 1 [file microorganisms-08-00736-s001.zip › Supp/Figure_S1.tif]
